# Resveratrol Enhances Palmitate-Induced ER Stress and Apoptosis in Cancer Cells

**DOI:** 10.1371/journal.pone.0113929

**Published:** 2014-12-01

**Authors:** Cristina Rojas, Belén Pan-Castillo, Cristina Valls, Gerard Pujadas, Santi Garcia-Vallve, Lluis Arola, Miquel Mulero

**Affiliations:** 1 Nutrigenomics Research Group, Department of Biochemistry and Biotechnology, Rovira i Virgili University, Tarragona, 43007, Spain; 2 Reproductive Biology and Gynecological Oncology Group, Center for Nanohealth, Institute of Life, Swansea University, Swansea, SA28PP, United Kingdom; 3 Centre Tecnològic de Nutrició i Salut (CTNS), TECNIO, CEICS, Reus, 43204, Spain; Complutense University, Spain

## Abstract

**Background:**

Palmitate, a saturated fatty acid (FA), is known to induce toxicity and cell death in various types of cells. Resveratrol (RSV) is able to prevent pathogenesis and/or decelerate the progression of a variety of diseases. Several *in vitro* and *in vivo* studies have also shown a protective effect of RSV on fat accumulation induced by FAs. Additionally, endoplasmic reticulum (ER) stress has recently been linked to cellular adipogenic responses. To address the hypothesis that the RSV effect on excessive fat accumulation promoted by elevated saturated FAs could be partially mediated by a reduction of ER stress, we studied the RSV action on experimentally induced ER stress using palmitate in several cancer cell lines.

**Principal Findings:**

We show that, unexpectedly, RSV promotes an amplification of palmitate toxicity and cell death and that this mechanism is likely due to a perturbation of palmitate accumulation in the triglyceride form and to a less important membrane fluidity variation. Additionally, RSV decreases radical oxygen species (ROS) generation in palmitate-treated cells but leads to enhanced X-box binding protein-1 (XBP1) splicing and C/EBP homologous protein (CHOP) expression. These molecular effects are induced simultaneously to caspase-3 cleavage, suggesting that RSV promotes palmitate lipoapoptosis primarily through an ER stress-dependent mechanism. Moreover, the lipotoxicity reversion induced by eicosapentaenoic acid (EPA) or by a liver X receptor (LXR) agonist reinforces the hypothesis that RSV-mediated inhibition of palmitate channeling into triglyceride pools could be a key factor in the aggravation of palmitate-induced cytotoxicity.

**Conclusions:**

Our results suggest that RSV exerts its cytotoxic role in cancer cells exposed to a saturated FA context primarily by triglyceride accumulation inhibition, probably leading to an intracellular palmitate accumulation that triggers a lipid-mediated cell death. Additionally, this cell death is promoted by ER stress through a CHOP-mediated apoptotic process and may represent a potential anticancer strategy.

## Introduction

Adipocytes have a unique capacity to store excess fatty acids (FAs) in the form of triglycerides in lipid droplets, whereas non-adipose tissues, such as the liver, have a limited capacity for lipid storage. An overload of FAs induce lipotoxicity and cell death in non-adipose cells, including cardiomyocytes, β-cells and hepatocytes [1]–[4]. High doses of saturated FAs, such as palmitate, can cause cellular damage and even cell death, whereas elevated concentrations of oleate and linoleate, which are unsaturated FAs, are better tolerated [1], [2]. Although the detailed mechanisms underlying FA-induced lipotoxicity remain inconclusive, it is generally accepted that reactive oxygen species (ROS) and endoplasmic reticulum (ER) stress are the major intracellular mechanisms involved [4]–[8].

The ER is the major site in the cell for protein folding and trafficking, and many cellular functions depend on this compartment. Failure of the ER's adaptive capacity is defined as ER stress, and cells display various adaptative responses to relieve this situation. The unfolded protein response (UPR) is the primary adaptative response to ER stress and intersects with many different inflammatory and stress signaling pathways [9], [10]. Monitoring of the ER lumen and signaling through the canonical branches of the UPR are mediated by the following three ER membrane-associated proteins: (**a**) PERK (PKR-like eukaryotic initiation factor 2a kinase); (**b**) IRE1 (inositol requiring enzyme 1); and (**c**) ATF6 (activating transcription factor-6). When ER stress is not resolved, the cell is functionally compromised and may undergo apoptosis. Currently, several pathways have been directly implicated in ER stress-induced apoptosis. For example, the transcription factor C/EBP homologous protein (CHOP) is induced by ER stress at the transcriptional level, which sensitizes cells to apoptosis by down-regulation of B-cell lymphoma 2 (Bcl-2) and activation of GADD34 and ERO1α [11], [12]. ER stress also activates IRE1 and PERK, which have been implicated in the activation of the pro-apoptotic c-Jun NH_2_-terminal kinase (JNK) [13], [14].

Several reports have studied the link between resveratrol (RSV) effects (in its protective or cytotoxic outcomes) and ER stress related factors as novel molecular targets for the action of polyphenols [15]–[18]. Additionally, many *in vitro* and *in vivo* studies have also shown a protective effect of RSV and other polyphenols on the liver fat accumulation induced by saturated FAs or a high fat diet [19]–[22]. Aside from these protective effects, RSV is able to inhibit tumor initiation, promotion and progression in a variety of cell culture systems and animal models by mechanisms that included cell cycle arrest, kinase pathways inhibition and apoptosis activation [23]–[26].

Interestingly, metabolic alterations, characterized by increased glycolysis and lipogenesis, are a hallmark of cancer cells [27], [28]. Therefore, actively proliferating cancer cells present not only quantitative changes in *de novo* lipid biosynthesis but also modifications in the lipid membrane composition, affecting membrane fluidity, signal transduction and gene expression [29], [30]. A wide variety of cancers present changes in the lipid membrane composition, which is primarily characterized by saturated FA and monounsaturated FA accumulation. This accumulation appears to be less due to an increased uptake of saturated FAs and monounsaturated FAs than to exacerbated synthesis of endogenous FAs [31], [32].

Additionally, saturated and unsaturated FAs differ significantly in their contribution to lipotoxicity. Previous studies with primary cell cultures and cancer cell lines have suggested that lipotoxicity from the accumulation of long chain FAs is specific for saturated FAs. This selectivity has been attributed to the generation of specific proapoptotic lipid species or signaling molecules in response to saturated but not unsaturated FAs [33]. The nature of these signals may differ across cell types but includes ROS generation, *de novo* ceramide synthesis, nitric oxide generation, decreases in phosphatidylinositol-3-kinase, and primary effects on the mitochondrial structure and function. Long chain FAs may also suppress anti apoptotic factors, such as Bcl-2 [33].

To test the hypothesis that RSV impairment of excessive fat accumulation induced by elevated saturated FAs could be partially mediated by a reduction in the ER stress response, we experimentally induced ER stress using palmitate in several cancer cell lines with or without RSV. Unexpectedly, sub-toxic RSV levels (25 µM) did not rescue cells from palmitate-induced ER-stress and lipoapoptosis. In contrast, we obtained the following: (**a**) a RSV mediated apoptosis only in the presence of the saturated FA, and (**b**) a strong promotion of the lipotoxicity by the concomitant increase in the FA amount. We characterized this RSV effect at the molecular level and found that the stearoyl-CoA desaturase 1 (SCD1) role (unsaturation enrichment) is likely related to this cellular “phenotype”, but mainly palmitate storage in triglyceride pools appears to be critically involved in the higher sensitivity of cancer cells to the palmitate-induced lipotoxicity. These results reveal a relatively unknown RSV cytotoxic mechanism that could be exploited to target apoptosis promotion in transformed cells.

## Results

### RSV induces ER stress in HepG2 cells

**Figure 1:**
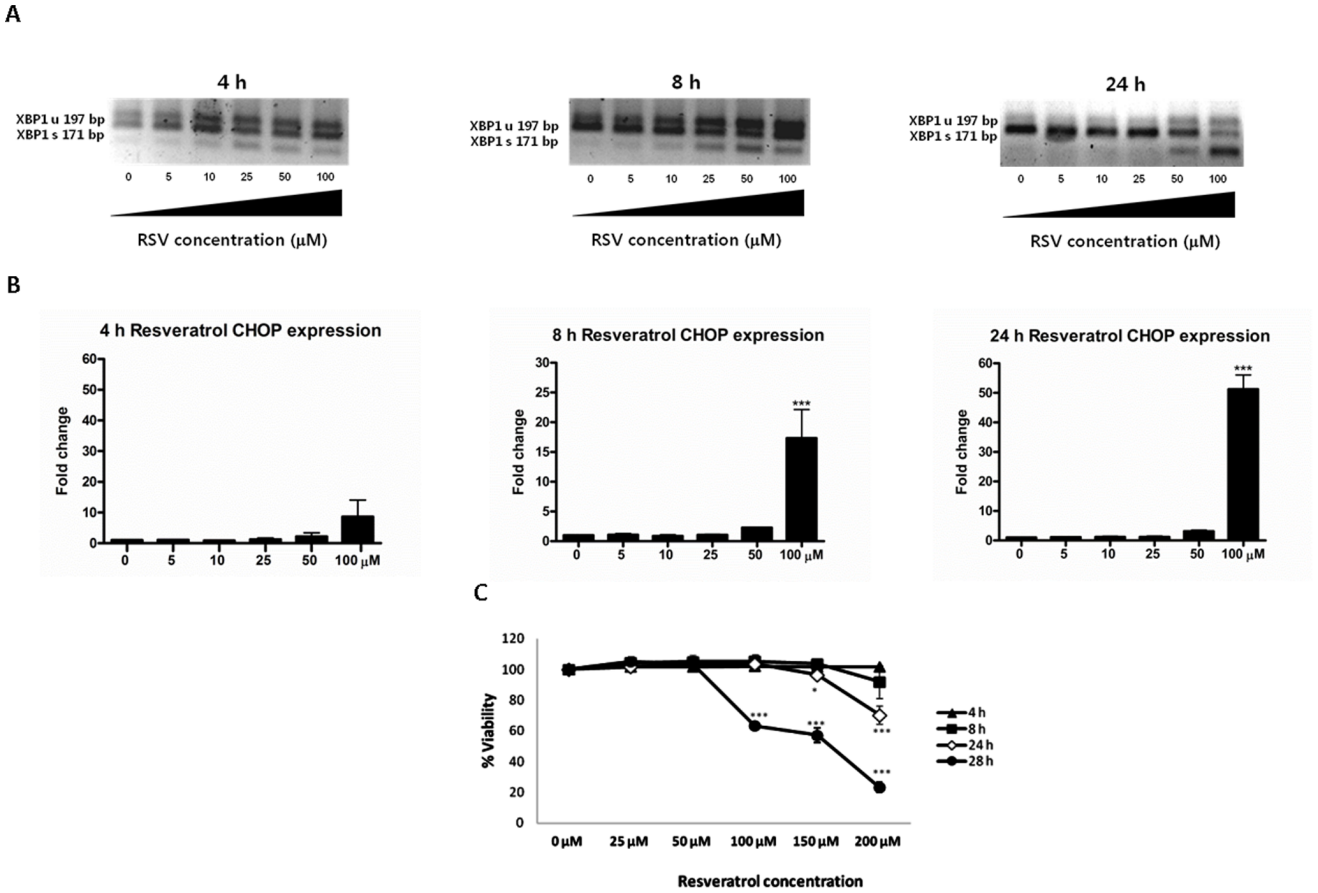
Time-dependent effects of increasing RSV concentrations on XBP1 splicing, CHOP expression and viability. HepG2 cells were exposed to vehicle (0 µM) or increasing RSV concentrations (5, 10, 25, 50 and 100 µM) and harvested at specific time points (8, 4 and 24 h). **A**) RSV exerts a time- and concentration-dependent activation of XBP1 splicing (XBP1 unspliced-197 bp amplicon; XBP1 spliced-171 bp amplicon). Representative image of three independent experiments **B**) RSV exerts a time- and concentration-dependent activation of CHOP expression. **C**) RSV decreases HepG2 viability at higher doses and incubation times. The viability was evaluated using a MTT assay. The data are shown as the mean ± SD of three independent experiments. Significant differences relative to the control (vehicle) were analyzed by one-way ANOVA followed by the Bonferroni post hoc test: ***p<0.001 and *p<0.05.

Figure **1** shows that HepG2 cells exposed to increasing RSV concentrations (ranging from 5 to 100 µM) for different durations (4, 8 and 24 h) have alterations in the ER homeostasis, consequently presenting active ER stress mechanisms. The detailed effect on X-box binding protein-1 (XBP1) splicing and CHOP expression was evaluated (figure **1A and 1B**). The maximal increase in XBP1 splicing (nearly all of the XBP1 in the spliced form) and in CHOP expression (51.29±4.81-fold change; p<0.001) was at a 100 µM RSV concentration and a 24 h incubation. Although the ER stress at 24 h is evident, there is a lack of correlation with cell viability, suggesting that although the cell is close to failing due to the ER malfunction, it remains viable; the decrease in viability appears after 24 h of RSV treatment (figure **1C**) with a value of ∼40% at 28 h (p<0.001). Note that the selected RSV concentration (25 µM) used in further experiments was unable to induce significant ER stress at any time point.

### RSV exacerbates palmitate-induced cell death in HepG2 cells

**Figure 2:**
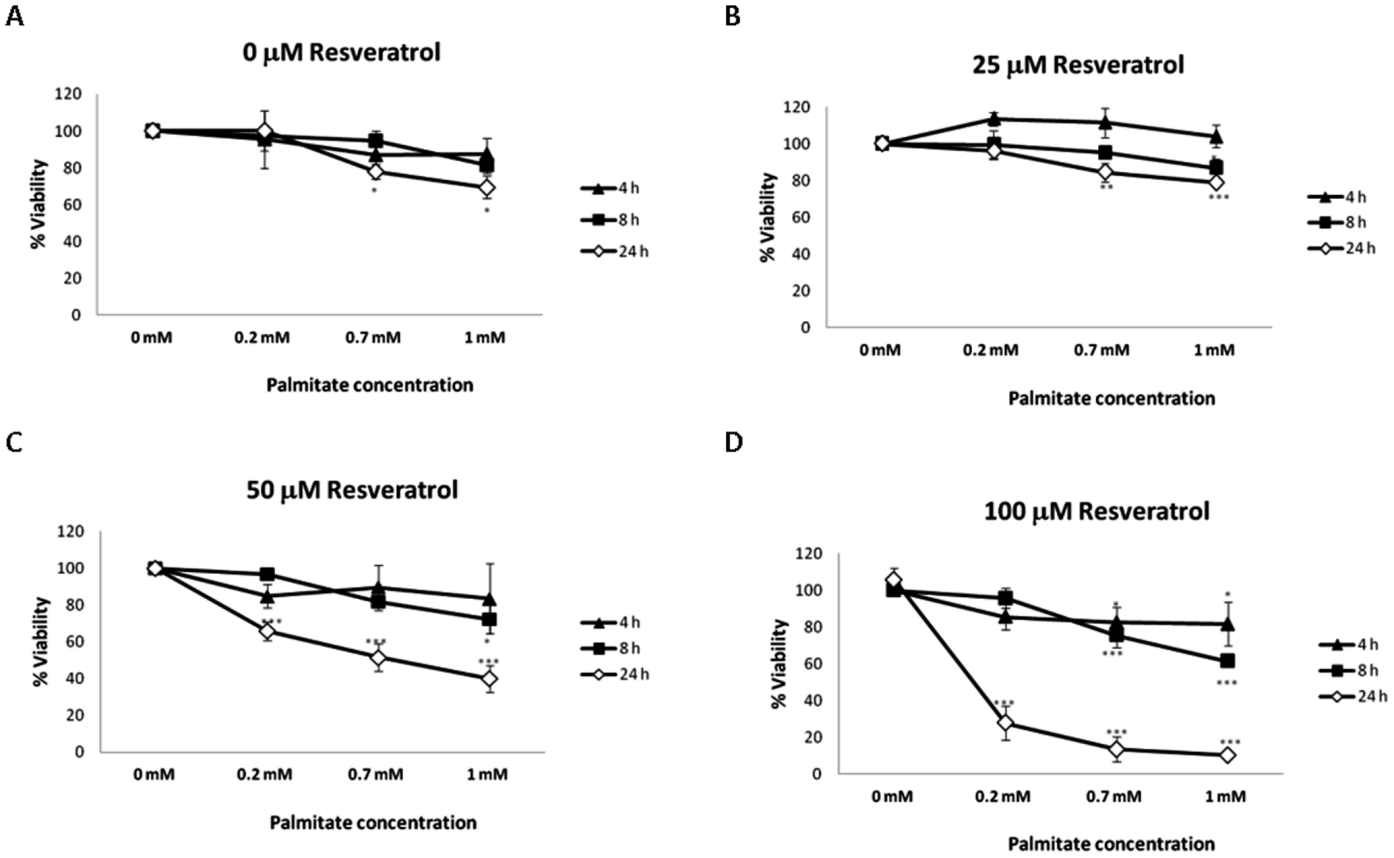
Palmitate-induced cell death is enhanced by RSV. Palmitate-induced cell death (expressed as the percentage of cellular viability) was evaluated by a MTT assay in HepG2 cells. The RSV effect on palmitate-treated cells was also evaluated. **A**) Increasing palmitate concentrations (ranging from 0.2 mM to 1 mM) caused a time- (4, 8 and 24 h) and dose-dependent decrease on cellular viability. Palmitate-treated cells were also co-incubated with increasing concentrations of RSV as follows: **B**) 25 µM RSV; **C**) 50 µM RSV and **D**) 100 µMRSV. A further decrease on HepG2 viability compared with treatments with palmitate alone was observed in the 50 µM RSV and 100 µM RSV assays. The data are expressed as the mean ± SD of three independent experiments. Significant differences relative to the control (vehicle) were analyzed by one-way ANOVA followed by the Bonferroni post hoc test: ***p<0.001 and *p<0.05.

Palmitate-induced cell death (expressed as a decrease in cellular viability) was evaluated by an MTT assay on the HepG2 cells. The RSV effect on palmitate-treated cells was also evaluated. As shown in figure **2A**, increasing concentrations of palmitate (ranging from 0.2 mM to 1 mM) caused a time- (4, 8 and 24 h) and dose-dependent decrease of cellular viability. When palmitate-treated cells were co-incubated with increasing RSV concentrations (ranging from 25 µM to 100 µM), a further decrease in the HepG2 viability was observed. This effect was more evident at 50 µM (∼60% maximal decrease of cellular viability; p<0.001) and 100 µM (∼80% maximal decrease of cellular viability; p<0.001) RSV treatments at 24 h of co-incubation (figure **2B-D**). Because of the lack of an additive effect of the 25 µM RSV concentration on palmitate-induced cell death (∼20% maximal decrease of cellular viability; n.s.), this concentration was selected to further study the RSV effect on ER stress and its relationship with fat accumulation induced by elevated FAs.

### RSV increases palmitate-induced ER stress in cancer cells

**Figure 3:**
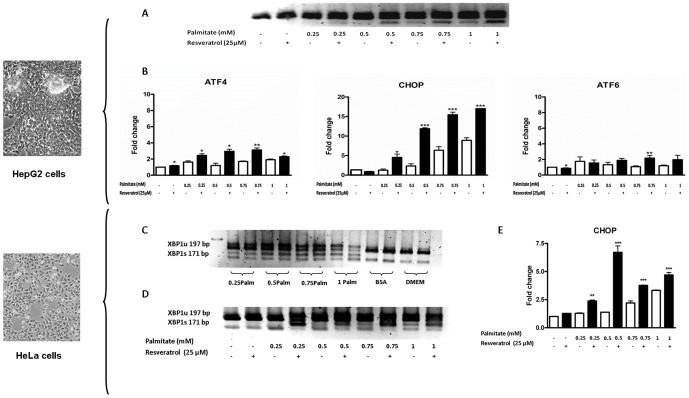
RSV increases palmitate-induced ER stress in cancer cells. HepG2 cells were washed twice in serum-free DMEM and transferred to serum-free medium with or without 25 µM RSV for 28 h; 8 h before the end of the experiment, increasing concentrations of palmitate (0.25, 0.5, 0.75 and 1 mM) were added to the corresponding well. **A**) XBP1 splicing. (XBP1 unspliced-197 bp amplicon; XBP1 spliced-171 bp amplicon). A representative image of three independent experiments is shown. **B**) ATF4, CHOP and ATF6 mRNA expression levels. The results are shown as the mean of the fold change ± SD of three independent experiments. HeLa cells were washed twice in serum-free DMEM and transferred to serum-free medium with or without 25 µM RSV for 28 h; 8 h before the end of the experiment, increasing concentrations of palmitate (0.25, 0.5, 0.75 and 1 mM) were added to the corresponding well. **C**) The susceptibility of HeLa cells to palmitate-induced ER stress. XBP1 splicing. (XBP1 unspliced-197 bp amplicon; XBP1 spliced-171 bp amplicon). A representative image of three independent experiments is shown. **D**) RSV exacerbates palmitate-induced splicing in HeLa cells. XBP1 splicing. (XBP1 unspliced-197 bp amplicon; XBP1 spliced-171 bp amplicon). A representative image of three independent experiments is shown **E**) RSV amplifies CHOP mediated signaling. CHOP mRNA expression levels. The results are shown as the mean of the fold change ± SD of three independent experiments. Significant differences relative to the control (vehicle) were analyzed by one-way ANOVA followed by the Bonferroni post hoc test: ***p<0.001 and **p<0.01.

The contribution of ER stress in palmitate-induced cell death was initially investigated using XBP1 splicing as an ER stress marker. Figure **3A** shows that a sub-toxic RSV concentration (25 µM) induced a concomitant increase in the palmitate effect on XBP1 splicing. To further verify our results, we studied other ER stress markers, such as ATF4, ATF6 and CHOP. Interestingly, the RSV effect was also observed (figure **3B**) on CHOP (17.03±0.01 vs. 8.91±0.65; p<0.001; the maximal fold change was between the RSV-treated and the non-treated samples) and ATF4 expression (3.13±0.35 vs. 1.7±0.09; p<0.01; the maximal fold change was between the RSV-treated and the non-treated samples). The effect was palmitate-dependent, indicating that although the same concentration of polyphenol was present, the stimulus that promoted a magnification of the molecular effects for nearly all of the studied ER stress markers was the FA elevation.

ATF6 was the only studied ER stress marker that appeared to be unaffected by the treatment. However, ATF6 translocation to the Golgi apparatus is required for its activation; therefore, it is likely that its expression is unaffected.

Globally, these results suggested that RSV promoted changes in several molecular mechanisms that were exacerbated when the amount of palmitate increased.

Remarkably, the same experimental result was obtained when another cancer cell line, HeLa cells, was used (figures **3C, 3D and 3E**). This suggests that this effect was not restricted to a specified cancer cell line.

### RSV sensitizes HepG2 cells to palmitate-induced apoptosis

To evaluate the RSV effect on palmitate lipoapoptosis, we developed Western blotting assays of cleaved caspase-3. The proapoptotic protein caspase-3 is synthesized as an inactive proenzyme (32 kDa) that is processed in cells undergoing apoptosis by self-proteolysis and/or cleavage by another upstream protease. The processed form of caspase-3 consists of large (17 kDa) and small (12 kDa) subunits that associate to form an active enzyme. The active caspase-3 proteolytically cleaves and activates other caspases and relevant targets in the cells, such as PARP and DFF [34].

**Figure 4:**
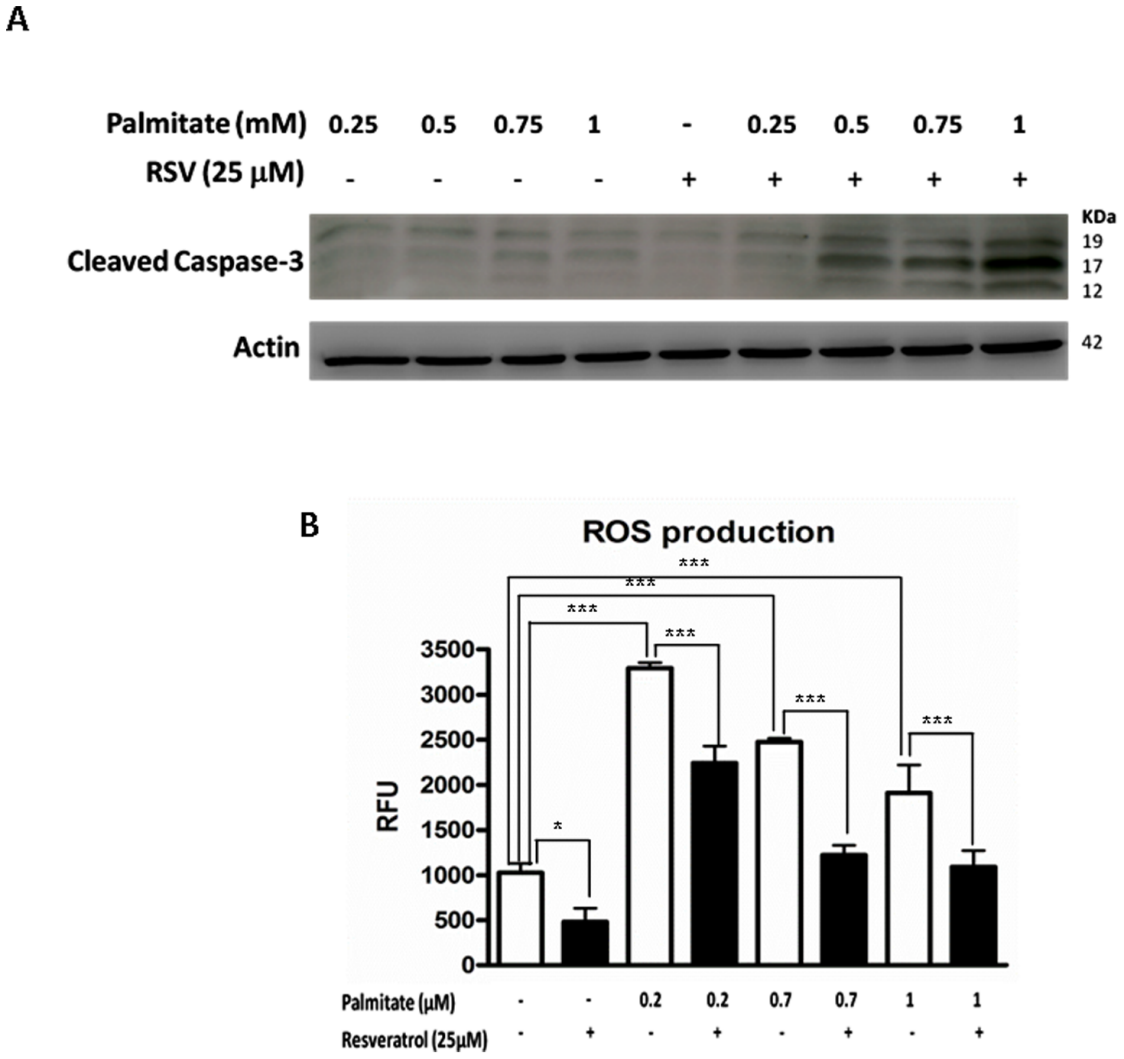
RSV increases apoptosis but decreases ROS production. HepG2 cells were washed twice in serum-free DMEM and transferred to serum-free medium with or without 25 µM RSV for 28 h; 8 h before the end of the experiment, increasing concentrations of palmitate (0.25, 0.5, 0.75 and 1 mM) were added to the corresponding well. **A**) Cleaved caspase-3 analysis by Western blotting. The blot shown is a representative image of three independent experiments. **B**) HepG2 cells were treated with or without 25 µM RSV for 28 h. Prior to an 8 h palmitate-RSV co-treatment, the cells were incubated with DCFH-DA (20 µM final concentration) at 37°C for 30 min. ROS production is shown as the mean of the RFU (Relative Fluorescence Units) ± SD of three independent experiments. Significant differences relative to the control (vehicle or palmitate) were analyzed by paired Student's t-tests. ***p<0.001,**p<0.01 and *p<0.05.

Figure **4A** shows that the palmitate treatments only induced a slight increase in the caspase-3 cleavage at higher doses (0.75 and 1 mM). When RSV was added, instead of reducing the apoptotic level, it promoted the lipoapoptotic effect of palmitate. Thus, RSV co-treatment induced the following: **(a)** augmentation of caspase-3 cleavage compared with that observed with palmitate alone; and **(b)** reduction of the saturated FA concentration threshold required to provoke the apoptotic effect (i.e., 0.5 mM palmitate vs. 0.5 mM palmitate +25 µM RSV).

### ROS production is reduced by RSV in palmitate-treated HepG2 cells

The contribution of oxidative stress in palmitate-induced cell death was investigated by detecting ROS production. For this assay, we measured the fluorescent signal after intracellular oxidation by ROS (such as hydrogen peroxide and hydroxyl radical) of the membrane permeable dye 2′,7′-dichloro-dihydro-fluorescein diacetate (DCFH-DA). Figure **4B** shows that culturing HepG2 cells with increasing concentrations of palmitate (ranging from 0.2 mM to 1 mM) for 8 h caused an increase in the fluorescent signal (1026±104 vs. 3294±61; p<0.001; the maximal difference was between the non-treated samples and the palmitate treated samples). Previous incubations with 25 µM RSV for 20 h decreased the amount of intracellular ROS in all of the assayed palmitate doses (2478±36 vs. 1222±108; p<0.001; the maximal difference was between the palmitate treated samples and the RSV + palmitate treated samples). This result supports the established antioxidant capacity of the polyphenol [35] and suggests that the aforementioned RSV effects related to the exacerbation of the palmitate effect on HepG2 cells are not primarily due to an increase in the intracellular ROS production.

### SCD1 dynamics are altered in response to RSV

**Figure 5:**
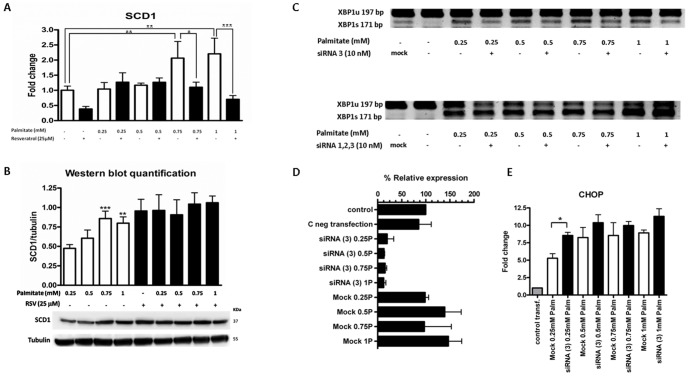
SCD1 dynamics are altered in response to RSV. HepG2 cells were washed twice in serum-free DMEM and transferred to serum-free medium with or without 25 µM RSV for 28 h; 8 h before the end of the experiment, increasing concentrations of palmitate (0.25, 0.5, 0.75 and 1 mM) were added to the corresponding well. **A**) SCD1 gene expression levels. The results are shown as the mean of the fold change ± SD of three independent experiments. **B**) SCD1 protein levels. The cell lysates were prepared and analyzed by Western blotting. The results of triplicate immunoblots were quantified by densitometry. The results shown in the graph represent the ratio of SCD1/tubulin. A representative immunoblot is shown below the graph. Significant differences relative to the control (vehicle or palmitate) were analyzed by paired Student's t-tests for SCD1 mRNA expression and by one-way ANOVA followed by the Bonferroni post hoc test for Western blot quantification.***p<0.001,**p<0.01 and *p<0.05. SCD1 knockdown (**C**) induces XBP1 splicing but this splicing is reduced when depleted SCD1 cells are exposed to increasing palmitate concentrations. (XBP1 unspliced-197 bp amplicon; XBP1 spliced-171 bp amplicon). A representative image of three independent experiments is shown. **D**) The decrease in XBP1 splicing in siRNA+palmitate cells is not due to SCD1 restoration. The percentage of relative SCD1 gene expression is shown. The results are represented as the mean of the fold change ± SD of three independent experiments. **E**) SCD1 silencing promotes a slight increase of CHOP expression. The results are shown as the mean of the fold change ± SD of three independent experiments.

It has been previously shown that among the nutritional stimuli that modulate SCD1 gene expression, saturated fats were strong activators. In cultured myotubes, palmitate increased SCD1 expression associated with an increase in the FA muscle storage [36]. Figure **5A** shows that 8 h treatment with increasing palmitate concentrations induced a significant overexpression of SCD1 at higher palmitate doses (1±0.14 vs. 2.21±0.52; p<0.001; the maximal fold change was between the palmitate treated and the non-treated samples). In contrast, when HepG2 cells were pre-treated for 20 h with RSV, SCD1 overexpression significantly decreased, suggesting that RSV impairs the palmitate-induced increase in SCD1 expression (2.21±0.52 vs. 0.7±0.12; p<0.001; the maximal difference was between the palmitate treated samples and the RSV + palmitate treated samples). Conversely, a slight decrease in the protein content was obtained when SCD1 was studied at the protein level (figure **5B**). This lack of correlation between the SCD1 mRNA and protein levels suggests that factors (post-translational factors, enzymatic activity regulation) other than gene expression could also be important to the SCD1 dynamics in response to RSV. Due to this discrepancy, we developed siRNA knockdown experiments to clarify the role of SCD1 in the RSV-induced ER stress. SCD1 expression significantly decreased due to siRNA transfection (figure **S1A**). The major levels of inhibition were obtained at a 10 nM siRNA concentration using siRNA-(3) or using a combination of the three siRNA oligonucleotides siRNA-(1, 2, and 3). Accordingly, SCD1 protein content (figure **S1B**) was also significantly reduced due to this experimental approach. Once the SCD1 knockdown was validated, we studied the effect of this gene silencing on the ER stress mechanisms using XBP1 splicing as an ER stress marker. Interestingly, as has been previously described by other authors [37], SCD1 inhibition activated XBP1 splicing (figure **S1C**), suggesting that the decrease in membrane unsaturation could trigger ER-stress and cell death. We selected siRNA (3) and the combination of the three siRNA oligonucleotides siRNA-(1, 2, and 3) to further study the effect of SCD1 inhibition in a saturated FA context (palmitate treatment). Surprisingly, both of the siRNA silencing approaches (figures **5C**) showed that the subsequent exposure of palmitate to experimentally SCD1-depleted cells did not exacerbate XBP1 splicing; conversely, in the presence of palmitate, this splicing was slightly decreased. To validate that this cellular phenotype was not due to a hypothetical “overriding” of the silencing strategy, we studied SCD1 expression once siRNA-(3) was transfected. The palmitate treatment was unable to reverse the SCD1 genetic suppression (figure **5D**). Therefore, as discussed below, other compensatory mechanisms promoted by the SCD1 inhibition could be responsible for the relative decrease in the XBP1 splicing. Additionally, figure **5E** shows that CHOP levels slightly increased because of SCD-1 inhibition, suggesting that despite the relative decrease in XBP1 splicing, other sensors of ER stress, such as ATF6 or PERK, could be influencing CHOP expression. Notably, this CHOP elevation is not comparable to that obtained previously with the RSV + palmitate treatments (figure **3B**).

### RSV inhibition of palmitate accumulation into triglyceride pools correlates with the increase in the CHOP and XBP-1 splicing

To elucidate other possible RSV molecular mechanism that promotes the exacerbation of ER stress-derived apoptosis, we focused our attention on triglyceride accumulation.

**Figure 6:**
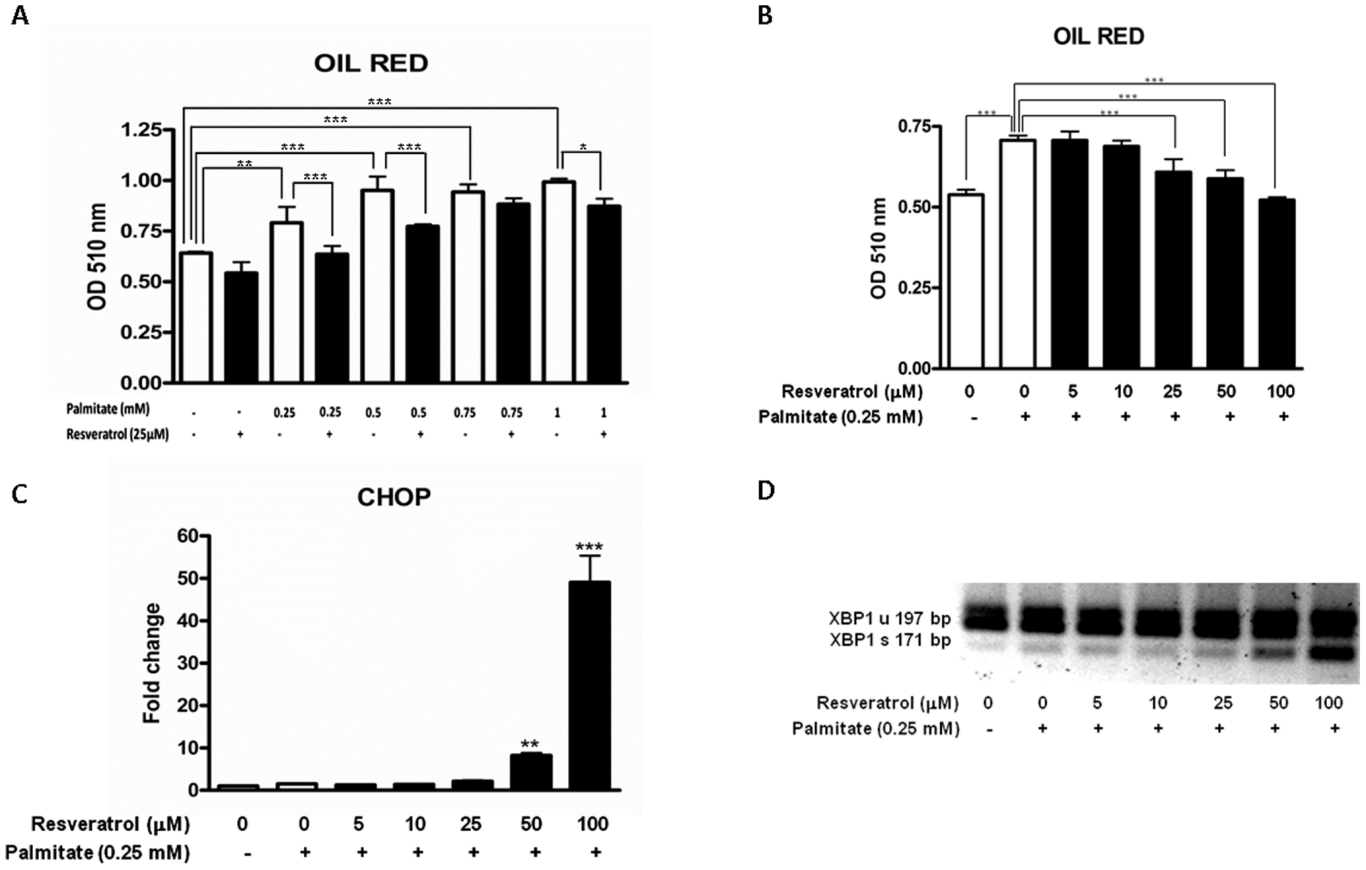
RSV inhibition of palmitate channeling into triglyceride pools correlates with the increase of CHOP and XBP-1 splicing. HepG2 cells were washed twice in serum-free DMEM and transferred to serum-free medium with or without 25 µM RSV for 28 h; 8 h before the end of the experiment, increasing concentrations of palmitate (0.25, 0.5, 0.75 and 1 mM) were added to the corresponding well. A) Oil red O staining quantification. Triglyceride accumulation is expressed as the mean of the OD (Optical density) ± SD of four independent experiments. For the fixed RSV concentrations, HepG2 cells were washed twice in serum-free DMEM and transferred to serum-free medium with a vehicle (0.1% ethanol), 5, 10, 25, 50 or 100 µM RSV and a fixed concentration of palmitate (0.25 mM) for 24 h. B) Oil red O staining quantification. Triglyceride accumulation is expressed as the mean of the OD (Optical density) ± SD of four independent experiments. Significant differences relative to the control (vehicle) and 0.25 mM palmitate were analyzed by one-way ANOVA followed by the Bonferroni post hoc test: ***p<0.001. C) CHOP mRNA expression levels. The results are shown as the mean of the fold change ± SD of three independent experiments. Significant differences relative to the control (vehicle) were analyzed by one-way ANOVA followed by the Bonferroni post hoc test: ***p<0.001 and **p<0.01. D) XBP1 splicing. (XBP1 unspliced-197 bp amplicon; XBP1 spliced-171 bp amplicon). A representative image of three independent experiments is shown.

Triglyceride storage was evaluated by oil red O staining. Figure **6A** shows that 8 h palmitate treatment induced a dose-dependent increase in HepG2 triglyceride content (0.643±0.006 vs. 0.994±0.015; p<0.001; the maximal difference was between the non-treated samples and the palmitate treated samples). Conversely, when cells were preincubated for 20 h with RSV, the intracellular lipid content was reduced at all of the assayed palmitate concentrations (0.951±0.068 vs. 0.774±0.011; p<0.001; the maximal difference was between the palmitate treated samples and the RSV + palmitate treated samples). These results are in accordance with the accumulated *in vitro* and *in vivo* evidence of the anti-adipogenic effect of RSV [38], [39].

Additionally, we developed several experiments in which we fixed the palmitate concentration (0.25 mM) and exposed HepG2 cells to increasing RSV concentrations. Notably, RSV was able to decrease triglyceride accumulation in a dose-dependent manner (figure **6B**). Despite this decrease in triglyceride accumulation, there was a concomitant activation of a primary ER stress component, such as XBP1 splicing and the ER-derived downstream apoptotic factor CHOP (figure **6C and 6D**). This result strongly suggested that, despite the initial beneficial effect of RSV in decreasing the triglyceride accumulation, there was a threshold of RSV-induced inhibition of lipid accumulation (25 µM RSV) that, once reached, triggered the ER stress-derived apoptosis induced by palmitate. Therefore, it appeared that, although the buffering capacity of palmitate by the cell is inhibited by RSV (primarily by the well-known RSV inhibitory effect of SREBP1c), when this inhibition is excessively strong/continuous, the amount of the remaining palmitate inside the cell will increase and promote the harmful effects of the saturated FA (palmitate-induced ER stress and apoptosis).

### Reversion of the RSV effects due to co-treatments with eicosapentaenoic acid (EPA) or the Liver X receptor (LXR) agonist (TO-901317)

**Figure 7:**
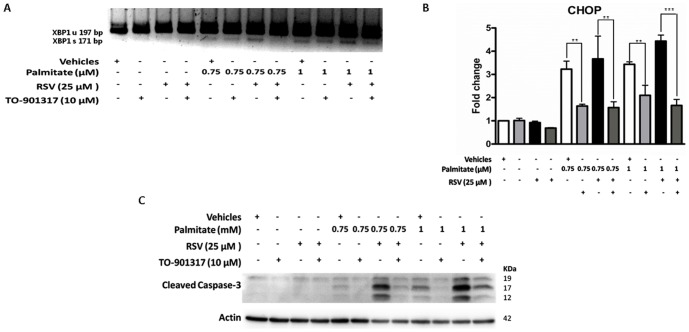
EPA rescued cells from RSV+palmitate induced ER stress and apoptosis. HepG2 cells were washed twice in serum-free DMEM and transferred to serum-free medium with a vehicle (0.1% ethanol), 25 µM RSV, 50 µM EPA or 100 µM EPA for 28 h; 8 h before the end of the experiment, increasing concentrations of palmitate (0, 0.75 and 1 mM) were added to the corresponding well. **A**) XBP1 splicing. (XBP1 unspliced-197 bp amplicon; XBP1 spliced-171 bp amplicon). A representative image of three independent experiments is shown. **B**) CHOP mRNA expression levels. The results are shown as the mean of the fold change ± SD of three independent experiments. **C**) The cleaved caspase-3 level was determined by Western blotting. The blot shown is a representative image of three independent experiments. Significant differences were analyzed by one-way ANOVA followed by the Bonferroni post hoc test: ***p<0.001 and **p<0.01.

To further examine whether ER stress induction in RSV + palmitate-treated cells is due to alterations in the palmitate processing capacity of the cell, we developed the following two experimental approaches: **(a)** polyunsaturated fatty acid (PUFA) supplementation (treatment with EPA at two increasing concentrations) and **(b)** LXR agonist treatment (addition of TO-901317 to stimulate SCD1 expression). Strikingly, figure **7C** shows that the supplementation of both of the EPA concentrations rescued HepG2 cells from the apoptotic process (reduced levels of cleaved caspase 3 compared with RSV + palmitate). This reduced level of the apoptotic factor correlated with a decrease in XBP1 splicing (figure **7A**) and CHOP expression (figure **7B**) (16.54±2.16 vs. 6.27±0.67; p<0.001; the maximal difference was between the RSV + palmitate treated samples and the RSV + palmitate + EPA treated samples), suggesting restoration of ER function.

**Figure 8:**
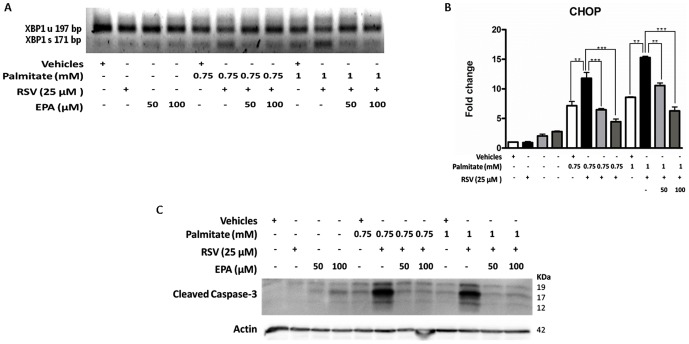
TO-901317 rescued cells from RSV+palmitate induced ER stress and apoptosis. HepG2 cells were washed twice in serum-free DMEM and transferred to serum-free medium with vehicles (0.05% ethanol and 0.1% DMSO) and 25 µM RSV or 10 mM TO-901317 for 28 h; 8 h before the end of the experiment, increasing concentrations of palmitate (0.75 and 1 mM) were added to the corresponding well. A) XBP1 splicing. (XBP1 unspliced-197 bp amplicon; XBP1 spliced-171 bp amplicon). A representative image of three independent experiments is shown. B) CHOP mRNA expression levels. The results are shown as the mean of the fold change ± SD of three independent experiments C) The cleaved caspase-3 level was determined by Western blotting. The blot shown is a representative image of three independent experiments. Significant differences were analyzed by one-way ANOVA followed by the Bonferroni post hoc test: ***p<0.001 and **p<0.01.

Alternatively, HepG2 cells treated with two concentrations (1 µM and 10 µM) of LXR agonist TO-901317 showed increased SCD1 protein and mRNA levels (figure **S2A** and **S2B**). Additionally, Figure **8** shows that agonist treatment also corrected the effects promoted by palmitate and triggered by RSV. For example, the agonist treatment corrected the increase in the caspase-3 cleavage (figure **8A**) and revoked the effects on ER stress (figure **8B and 8C**) in XBP1 and in CHOP expression (4.43±0.26 vs. 1.67±0.26; p<0.001; the maximal difference was between the RSV + palmitate treated samples and the RSV + palmitate + TO-901317 treated samples).

## Discussion

The cell-protective features of the ER stress response appear to be chronically activated in tumor cells, thus providing support for continuous proliferation and survival, even under adverse microenvironmental conditions [40], [41]. However, the persistent activity of these pro-survival pathways primarily in tumor cells may provide a window of opportunity for therapeutic intervention that is principally aimed at these tumor-specific conditions. Accordingly, appropriate therapeutic regimens would seek to further aggravate this already engaged system in tumor cells to exhaust its protective features and, instead, trigger its pro-apoptotic module [42]. Interestingly, here we show for the first time that an interaction between a polyphenol and a saturated FA could “take profit” of this window of opportunity and induce a potent ER-mediated cytotoxic effect in several cancer cell lines. And, that this fact is likely due to the RSV-mediated perturbation of palmitate managing in cancer cells.

In this sense, despite previous studies have shown that RSV is able to reduce the triglyceride content in palmitate-treated cells and in animals and that this effect is mediated by the inhibition of SREBP1c expression via Sirt-1-FOXO1 signaling pathways [20], [21], none of them have focused on the possible cytotoxic outcome of such intervention. Interestingly, we have also observed this previously described anti-adipogenic RSV effect (figure **6A**), but when the FA concentration is fixed, the decrease in the triglyceride accumulation is strongly correlated with a significant increase in XBP1 splicing and CHOP expression (figure **6B, 6C and 6D**). It has been previously shown that when cultured cells are exposed to high concentrations of palmitate for up to 24 h, triglyceride synthesis prevents lipotoxicity [33]. It appears that, in this context, the palmitate is channeled toward triglyceride storage and is rendered unavailable for pathways leading to cell death, such as the generation of ROS and ceramide. Therefore, it is feasible that RSV could increase the lipotoxic effect by avoiding palmitate storage in triglyceride pools, allowing the detrimental effect of these saturated FAs that will finally promote an indirect RSV-induced ER stress. Additionally, our results suggest that due to the antioxidant nature of the polyphenol, the lipotoxic effect could hypothetically be mediated by ceramide formation.

A wide variety of cancers present changes in the lipid membrane composition. Although the overexpression of acetyl Co-A carboxylase and FA synthase have been described in various cancers [43], [44], an increased monounsaturated FAs content could also be associated with overexpression of SCD1 [45]. Specifically, SCD1 is a 40 kDa intrinsic membrane protein anchored in the ER. This iron-containing enzyme catalyzes the biosynthesis of monounsaturated FAs [46]. SCD1 introduces a *cis* double bond in the Δ9 position of several saturated FAs, such as palmitic (16:0) and stearic (18:0) acids, to yield palmitoleic (16:1) and oleic (18:1) acids, respectively [47]. Interestingly, it has been found that SCD1 knockdown in HeLa cells led to increases in the saturated FAs, 16:0 and 18:0, and decreases in the monounsaturated FAs, 16:1n-7, 18:1n-9, and 18:1n-7 in phospholipids, which leads to a decrease in membrane phospholipid unsaturation and death [48]. Additionally, it has been also shown that the inhibition of SCD1 expression induces CHOP-dependent cell death in human cancer cells [37]. Therefore, the elucidation of other possible RSV effects led us to focus on the saturated FA *vs.* monounsaturated FA membrane ratio and, more concisely, on factors that could modulate this ratio such as SCD1. Our results indicate that RSV impaired palmitate-induced SCD1 mRNA overexpression at higher doses (0,75 and 1 mM palmitate). In agreement with our results, Ajmo and collaborators [22], while developing *in vivo* animal experiments to test the ability of RSV to reverse the inhibitory effects of chronic ethanol feeding and the prevention of alcoholic liver steatosis, have also shown that RSV (200 and 400 mg/kg body weight) reduced the SCD1 mRNA level, even in control mice.

Surprisingly, only slight changes were observed when SCD1 protein content was studied. This result could suggest that RSV targets SCD1 not only at a transcriptional level but also at a post-transductional level. Nevertheless, to provide evidence for the direct role of SCD1 on the observed cellular “phenotype”, a silencing experimental approach was developed (siRNA silencing). The results clearly show that SCD1 genetic ablation in the presence of the saturated FA does not provide the same experimental results on XBP1 splicing or on CHOP expression compared with that obtained with RSV. On the contrary, the absence of SCD1 triggers ER stress, but the subsequent palmitate addition decreases such stress. This is an interesting result because it means that: (**a**) despite decreasing SCD1 mRNA levels, RSV is not exerting its effect exclusively through SCD1 extinction, and (**b**) SCD1 silencing is a good cytotoxic strategy only in the absence of excessive saturated FA. The explanation of the last point is beyond the scope of this paper, but we can speculate about the reason of such surprising result. For example, Thörn and co-authors [49] found that the knockdown of SCD1 mainly up-regulated the proteins involved in protein folding and degradation, and this could be one possibility of why a subsequent palmitate exposure is not increasing XBP1 splicing.

Importantly, the idea that RSV + palmitate critically hinders the cell's capacity to mediate palmitate, consequently promoting UPR, was further supported by the observation that the lipotoxicity could be effectively reversed by ω-3 fatty acid supplementation using EPA or by treatment with the LXR agonist TO-901317.

On the one hand, there are several mechanisms through which unsaturated FAs, such as EPA, may promote triglyceride accumulation, as follows: (**a**) unsaturated FAs can serve as ligands for transcription factors, such as peroxisome proliferator activated receptor gamma, (**b**) the possible activation of signaling pathways that promote triglyceride storage (or inhibit triglyceride hydrolysis) by unsaturated FAs, and (**c**) the increased solubility/stability of lipid droplets containing a higher percentage of unsaturated acyl-chains.

On the other hand, in the case of the LXR agonist treatment (TO-901317), it is feasible that the upregulation of SREBP1c counteracts the RSV inhibitory effect and stimulates the adipogenic response; and/or the presence of increased quantities of endogenous monounsaturated FAs due to SCD1 overexpression, such as palmitoleoyl–CoA, could facilitate the accumulation of saturated FAs in the triglyceride stores. Interestingly, it has been shown that SCD1 inhibition causes cancer cell death by depleting monounsaturated FAs [50].

However, although we showed that an important part of the RSV effect could be mediated by a modulation on the lipogenic response, Borradaile and collaborators [51] have reported that administered palmitate is rapidly incorporated into lipid components of the ER and impairs the ER structure and integrity, suggesting that the ER membrane plays an important proximal role in palmitate-induced toxicity by ER stress. Nevertheless, the results obtained by fluorescence quenching and anisotropy studies indicate that RSV has a membrane fluidizing effect and is able to permeate the membrane, even in the gel phase [52]. This result suggests that the hypothetical direct membrane rigidification induced by palmitate could be, at least partially, counteracted by RSV. Further experiments are required to corroborate this hypothesis.

Although we have not yet developed a primary hepatocytes culture to test the RSV effect on non-transformed cells exposed to increasing palmitate doses, other authors [37] have shown that normal and cancer cells do not respond in the same manner to the prevention of MUFA synthesis by siRNA-mediated SCD1 extinction. These authors have observed that cancer cells were killed by SCD1 depletion, whereas non-cancer cells remained alive, suggesting that the viability of non-cancer cells remained unaffected because they do not require such rapid and high MUFA synthesis.

Finally, although RSV alone is able to induce ER stress at high doses (such as 100 µM), it also has subtle effects at low doses (25 µM). Importantly, these effects could be used to promote an apoptotic cell death by palmitate overload in cancer cells. These results have potential practical implications in the following aspects: **(a)** they suggest that this additive effect could be exploited to target the low bioavailability of RSV [53], [54] because it is possible to promote a RSV-associated toxicity in cancer cells when the transformed cells are also exposed to a richly saturated FA environment, and **(b)** they highlight that RSV-mediated inhibition of lipogenesis in a saturated fatty acid context could represent a promising anticancer therapy by inducing cell death through ER stress and CHOP activation.

## Materials and Methods

### Chemicals

Bovine Serum Albumin (BSA) ref. A8806, sodium palmitate ref. P9767, resveratrol (RSV) ref. R5010, cis-5,8,11,14,17–eicosapentaenoic acid (EPA) ref. E2011, TO-901517 ref. T2320, Thiazolyl Blue Tetra-zolium Bromide (MTT) ref. M5655, Oil Red O O0625 and 2′,7′-dichlorofluorescein-diacetate (DCFH-DA) ref. 35845, the protease inhibitor cocktail ref. P8340, the phosphatase inhibitor cocktail 2 ref. P5726 and the phosphatase inhibitor cocktail 3 ref. P0044 were obtained from Sigma-Aldrich (Sr.Louis,USA). Dulbecco's modified Eagle's medium (DMEM) was purchased from Lonza (Basel, Switzerland). The human hepatoblastoma HepG2 cell line was obtained from the European Collection of Cell Cultures (Wiltshire,UK).

### Cell culture and general experimental treatment

The human hepatoblastoma HepG2 cells were cultured in 75 cm^2^ flasks (Orange Scientific) with DMEM supplemented with 10% fetal bovine serum, 2% PS (penicillin-streptomycin), 1% L-glutamine, and 1% NEAA (non-essential amino acids) in a humidified atmosphere with 5% CO_2_ at 37°C. For the mRNA and protein extraction, the cells were seeded at a density of 5×10^5^ cells/well in 12-well plates (Orange Scientific). The RSV treatments were developed by incubating the HepG2 cells with increasing concentrations of polyphenol (0, 5, 10, 25, 50 or 100 µM) or a vehicle (0.1% ethanol) and harvested at specific time points (8, 4 and 24 h). For the palmitate-RSV treatments, the HepG2 cells were washed twice in serum-free DMEM and transferred to serum-free medium with or without 25 µM RSV for 28 h; 8 h before the end of the experiment, increasing concentrations of palmitate (0.25, 0.5, 0.75 and 1 mM) were added to the corresponding well using albumin as fatty acid carrier (7:1 palmitate-to-albumin ratio). For the unsaturation enrichment experiments, EPA (50 or 100 µM) or TO-901317 (10 µM) was added concomitant with the palmitate.

### Palmitate-BSA solution preparation

The Palmitate-BSA complexes were prepared as previously described [55] with minor modifications. Briefly, 13.9 mg sodium palmitate was dissolved in 0.5 mL sterile water (100 mM palmitate stock solution) by heating (70°C) and mixing (250 rpm) for 10 min in a thermomixer (Grant-Bio). A portion of the palmitate stock solution (50 µL) was added to 950 µL serum-free DMEM containing 5% NEFA-free BSA (the 5 mM palmitate working solution). The palmitate working solution was also heated (40°C) and shaken (250 rpm) for 1 h. Finally, the working solution was filtered (20 nm diameter filter) and immediately used to treat the cells. Serum-free DMEM containing 5% NEFA-free BSA was used as the control vehicle.

### MTT assay

The MTT colorimetric assay determined the ability of the viable cells to convert a soluble tetrazolium salt [3–(4,5-dimethylthiazol-2-yl)-2,5-diphenyltetrazolium bromide] (MTT) into an insoluble formazan precipitate. Tetrazolium salts accept electrons from oxidized substrates or appropriate enzymes, such as NADH and NADPH. In particular, MTT is reduced at the ubiquinone and cytochrome b and c sites of the mitochondrial electron transport system and is the result of succinate dehydrogenase activity. This reaction converts the yellow salts to blue-colored formazan crystals that can be dissolved in an organic solvent whose concentration can be spectrophotometrically determined.

For this assay, cells were seeded at a density of 5×10^4^ cells/well on a 96-well culture plate and incubated overnight. After the treatments, the medium was removed and was replaced with 200 µL fresh medium. Cells were loaded with 50 µl freshly prepared MTT (5 mg/mL in phosphate buffered saline (PBS)) and incubated for 4 h at 37°C. The blue formazan crystals formed following the reduction of the MTT dye were solubilized in 200 µl dimethyl sulfoxide (DMSO) and 25 µl glycine buffer and quantified using the Helios Zeta UV-VIS ELISA reader (Thermo Scientific). The dye absorbance was measured at a wavelength of 570 nm (680 nm was used as the reference wavelength). The vehicle-treated cells were established as 100% viability. The relative percentage of viability was calculated as follows: Viability (%)  = [A570 (compound)/A570 (control)]×100. Cell survival or viability (%) was determined by averaging three repeated experiments.

### RNA isolation and cDNA synthesis

The total RNA was obtained from the HepG2 cells using a RNeasy Mini Kit (Qiagen, Valencia, CA) according to the manufacturer's protocol. The RNA was resuspended in 100 µL RNasefree water. The DNase I RNAase free kit (Fermentas, Thermo Scientific) was used to remove the genomic DNA from the RNA preparations. The RNA was quantified with a spectrophotometer (Nanodrop 1000 Spectrophotometer, Thermo Scientific) at an absorbance of 260 nm and tested for purity (by the A260/280 ratio) and integrity (by denaturing gel electrophoresis). The first strand of cDNA was reverse transcribed from 1 µg total RNA from each sample using a First Strand cDNA Synthesis Kit (Fermentas, Thermo Scientific) according to the manufacturer's protocol. An identical reaction without the reverse transcription was performed to verify the absence of genomic DNA. The cDNA was subsequently amplified by PCR using human-specific primers for SCD1 (forward: 5′-CCG ACG TGG CTT TTT CTT C-3′; reverse: 5′-CCT CCT CTG GAA CAT CAC CA-3′), CHOP (forward: 5′-AGG GAG AAC CAG GAA ACG GAA ACA-3′; reverse: 5′-TCC TGC TTG AGC CGT TCA TTC TCT-3′), ATF6 (forward: 5′-ATG TCT CCC CTT TCC TTA TAT GGT; reverse: 5′-AAG GCT TGG GCT GAA TTG AA-3′), ATF4 (forward: 5′-GGG TTC TCC AGC GAC AAG GCT AAG-3′; reverse: 5′-AAC AGG GCA TCC AAG TCG AAC TC-3′), and cyclophilin (forward: 5′-TTC ATC TGC ACT GCC AAG AC-3′; reverse: 5′-TCG AGT TGT CCA CAG TAG C-3′).

### Real-time RT-PCR

Quantitative PCR for CHOP, ATF6, ATF4 and cyclophilin was performed using SYBR Premix Ex Taq (Takara) according to the manufacturer's protocol and was analyzed on a CFX96 Real-Time PCR Detection System (BIORAD, Spain.). The thermal cycling was composed of an initial step at 50°C for 2 min followed by a polymerase activation step at 95°C for 10 min and a cycling step with the following conditions: 40 cycles of denaturation at 95°C for 15 s, annealing at 60°C for 1 min, and extension at 72°C for 1 min. Oligonucleotides of varying lengths produce dissociation peaks at different melting temperatures. Therefore, at the end of the PCR cycles, the PCR products were analyzed using a heat dissociation protocol to confirm that a single PCR product was detected by the SYBR Green dye. The fluorescence data were acquired at the 72°C step. The threshold cycle (Ct) was calculated using the CFX Manager Software to indicate significant fluorescence signals above the noise during the early cycles of amplification. The software calculated copy numbers for the target samples from the Ct using interpolation from the standard curve. The relative levels of expression of the target genes were measured using cyclophilin mRNA as an internal control according to the 2−ΔΔCt method.

### Analysis of XBP1 mRNA splicing

Spliced XBP1 mRNA induced by activated IRE1 is translated to the protein, a potent transcription factor that induces BiP/GRP78 expression. XBP1 splicing is also induced by activated ATF6; thus, it is believed to be an important marker reflecting IRE1 and ATF6 signaling in response to ER stress. For this assay, the XBP1 cDNAs were amplified by PCR using human-specific primers for the XBP1 transcript (forward: 5′-GCT GAA GAG GAG GCG GAA G-3′; reverse: 5′-GTC CAG AAT GCC CAA CAG G-3′). These primers are useful for capturing the XBP1 spliced forms (XBP1s-172 bp amplicon) and the XBP1 unspliced form (XBP1u-197 bp amplicon). The PCR conditions were composed of an initial step at 50°C for 2 min followed by a polymerase activation step at 95°C for 10 min and a cycling step with the following conditions: 40 cycles of denaturation at 95°C for 30 s, annealing at 54°C for 30 sec, and extension at 72°C for 30 sec. A final extension at 72°C for 10 min was also developed. The PCR products were separated by 4% agarose gel electrophoresis for 280 min and were stained with ethidium bromide.

### Oil red O staining

The HepG2 cells were grown on 12-well plates. After the treatment incubation, the plates were washed three times with PBS and fixed with 10% formaldehyde for 15 min at room temperature. After fixation, the cells were stained with a filtered oil red O working solution (stock solution, 5 mg/mL oil red O powder in isopropanol; working solution, 60% oil red O stock solution in distilled water) for 45 min at room temperature. The cells were then washed twice with PBS to remove unbound dye and were visualized under a microscope. After the microscopic examination, the amount of triglyceride was quantified in each well. Isopropanol (200 µL/well) was added to the staining plates, and the plates were shaken at room temperature for 5 min. The extracted dye was removed by gentle pipetting, and its absorbance was read spectrophotometrically at 510 nm.

### Measurement of intracellular reactive oxygen species (ROS) production

The intracellular formation of ROS (Reactive Oxygen Species) was detected using the fluorescent probe 2′,7′-dichlorofluorescein-diacetate (DCFH-DA). DCFH-DA diffuses into cells and is deacetylated by cellular esterases into the non-fluorescent DCFH. In the presence of ROS, DCFH is rapidly oxidized to highly fluorescent DCF.

The HepG2 cells were grown to 70-80% confluence in black polystyrene 96-well plates. The HepG2 cells were preincubated with or without 25 µM RSV in serum-free medium for 28 h. Prior to an 8 h palmitate-RSV co-treatment, the cells were incubated with DCFH-DA (20 µM final concentration) at 37°C for 30 min. The fluorescence was analyzed using a 37°C pre-heated plate reader (FLx800TBID, Biotek Instruments) using excitation and emission wavelengths of 475 and 525 nm, respectively. The ROS production was expressed in relative fluorescence units (RFU).

### Western blotting analysis

The HepG2 cells were harvested and homogenized in RIPA lysis buffer (50 mM Tris-Cl pH 7.4, 150 mM NaCl, 1% NP40, and 0.25% Na-deoxycholate, containing protease and phosphatase inhibitors). Aliquots of the cell lysate containing 30 µg of protein per sample were analyzed by Western blotting. Briefly, the samples were placed in sample buffer (0.5 M Tris–HCl pH 6.8, 10% glycerol, 2% (w/v) SDS, 5% (v/v) 2-β-mercaptoethanol, and 0.05% bromophenol blue) and denatured by boiling at 95–100°C for 5 min. The samples were then separated by electrophoresis on 15% (for the cleaved caspase-3 Western blotting) or 12% (for the SCD1 Western blotting) acrylamide gels. The proteins were subsequently transferred to polyvinylidene difluoride (PVDF) membranes (Amersham Biosciences, GE Healthcare) using a transblot apparatus (BioRad). The membranes were blocked for 1 h with 5% non-fat milk dissolved in TBS-T buffer (50 mM Tris, 1.5% NaCl, and 0.2% Tween 20, pH 7.5). The membranes were then incubated overnight with primary monoclonal antibodies against cleaved caspase-3 (Asp 175; 5A1E; Cell Signal), SCD1 (ab19862; Abcam), β-actin (A 2066; Sigma), or tubulin (T 3526; Sigma). The blots were washed thoroughly in TBS-T buffer and incubated for 1 h with a peroxidase-conjugated IgG antibody. The immunoreactive proteins were visualized using an enhanced chemiluminescence substrate kit (ECL plus; Amersham Biosciences, GE Healthcare) according to the manufacturer's instructions. Digital images were obtained with a GBOX Chemi XL 1.4 system (Syngene, UK), which permits quantification of the band intensity. The protein load was monitored via the immuno-detection of actin or tubulin.

### siRNA knockdown of SCD1 in the HepG2 cells

RNA interference to reduce SCD1 expression was performed with a set of three siRNA oligonucleotides (Trilencer-27 human siRNA) obtained from Origene Technologies Inc. (Rockville, MD, USA) (SR304248). The trilencer-27 universal scrambled negative control siRNA duplex (SR30004, OriGene) was used as the scrambled siRNA control (mock). The HepG2 cells were transfected for 24 h with increasing concentrations (0.1 nM, 1 nM and 10 nM) of each siRNA alone or in combination (SR304248A (1), SR304248B (2) and SR304248C (3)) using the siTRAN transfection reagent (Origene) following the manufacturer's protocol.

The total cell mRNA and the protein from these transfected cells were obtained and analyzed for SCD1 knockdown by real-time RT-PCR and Western blotting as previously described.

### Data analysis and Statistics (Statistical analysis)

The data were evaluated by Student's T-test or one-way ANOVA, followed by the Bonferroni post hoc tests to identify significant differences between the controls and the treatments; the Graphpad Prism version 4 software was used. The differences were considered significant when the P values were less than 0.05. The results are displayed as the mean±SD of at least three independent assays for each experiment.

## Supporting Information

Figure S1 **siRNA knockdown of SCD1.** A 10 nM siRNA concentration was effective on the SCD1 silencing for the majority of the siRNA used, as follows: alone (2 and 3) or in combination (1, 2 and 3). HepG2 cells were transfected for 24 h with increasing concentrations (0.1 nM, 1 nM and 10 nM) of Origene's siRNA alone or in combination (SR304248A (1), SR304248B (2) and SR304248C (3)) using the siTRAN transfection reagent (Origene). The total cell mRNA and the protein from these transfected cells were obtained and analyzed for SCD1 knockdown. **A)** Percentage of relative SCD1 gene expression. The results are shown as the mean of the fold change ± SD of three independent experiments. **B)** SCD1 protein levels. Cell lysates were prepared and analyzed by Western blotting. A representative immunoblot of three independent experiments is shown. **C)** SCD1 silencing induces XBP1 splicing. (XBP1 unspliced-197 bp amplicon; XBP1 spliced-171 bp amplicon). A representative image of three independent experiments is shown.
(TIF)

Figure S2 **The LXR activator TO-901317 induces SCD1 mRNA and protein expression.** HepG2 cells were treated with 0 (0.1% DMSO) and 1 or 10 µM TO-901317 for 20 h. A) SCD1 protein levels. Cell lysates were prepared and analyzed by Western blotting. The results of triplicate immunoblots were quantified by densitometry. The results shown in the graph represent the ratio of SCD1/tubulin. A representative immunoblot is shown below the graph. B) SCD1 gene expression levels. The results are shown as the mean of the fold change ± SD of three independent experiments. Significant differences relative to the control (vehicle) were analyzed by one-way ANOVA followed by the Bonferroni post hoc test for Western blot quantification.**p<0.01 and *p<0.05.
(TIF)
