# Supplementary figures and images for: Resveratrol Enhances Palmitate-Induced ER Stress and Apoptosis in Cancer Cells
Source: PLoS One. 2014 Dec 1;9(12):e113929. doi: 10.1371/journal.pone.0113929 (PMC4250062; doi:10.1371/journal.pone.0113929)

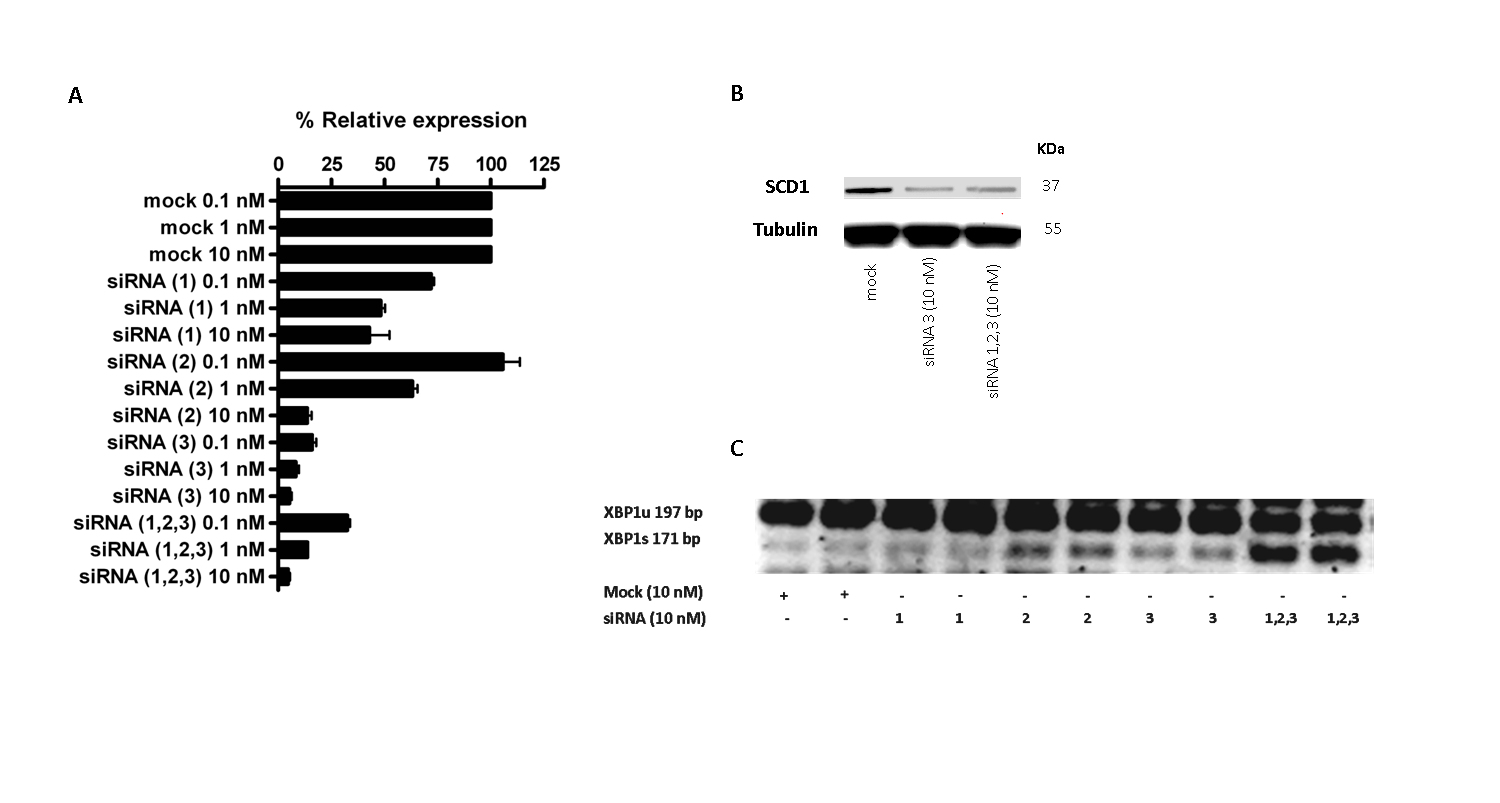

Supplement: Figure S1 — siRNA knockdown of SCD1. A 10 nM siRNA concentration was effective on the SCD1 silencing for the majority of the siRNA used, as follows: alone (2 and 3) or in combination (1, 2 and 3). HepG2 cells were transfected for 24 h with increasing concentrations (0.1 nM, 1 nM and 10 nM) of Origene's siRNA alone or in combination (SR304248A (1), SR304248B (2) and SR304248C (3)) using the siTRAN transfection reagent (Origene). The total cell mRNA and the protein from these transfected cells were obtained and analyzed for SCD1 knockdown. A) Percentage of relative SCD1 gene expression. The results are shown as the mean of the fold change ± SD of three independent experiments. B) SCD1 protein levels. Cell lysates were prepared and analyzed by Western blotting. A representative immunoblot of three independent experiments is shown. C) SCD1 silencing induces XBP1 splicing. (XBP1 unspliced-197 bp amplicon; XBP1 spliced-171 bp amplicon). A representative image of three independent experiments is shown. (TIF) [file pone.0113929.s001.tif]

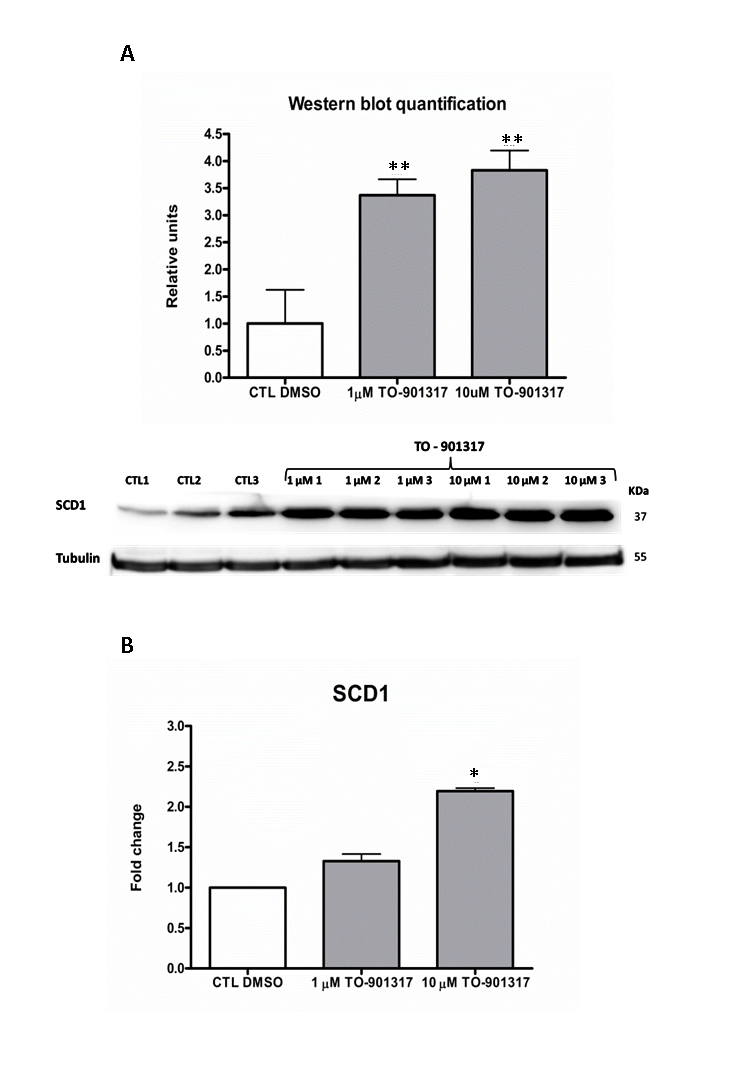

Supplement: Figure S2 — The LXR activator TO-901317 induces SCD1 mRNA and protein expression. HepG2 cells were treated with 0 (0.1% DMSO) and 1 or 10 µM TO-901317 for 20 h. A) SCD1 protein levels. Cell lysates were prepared and analyzed by Western blotting. The results of triplicate immunoblots were quantified by densitometry. The results shown in the graph represent the ratio of SCD1/tubulin. A representative immunoblot is shown below the graph. B) SCD1 gene expression levels. The results are shown as the mean of the fold change ± SD of three independent experiments. Significant differences relative to the control (vehicle) were analyzed by one-way ANOVA followed by the Bonferroni post hoc test for Western blot quantification.**p<0.01 and *p<0.05. (TIF) [file pone.0113929.s002.tif]
